# Acquired perforating collagenosis associated with ranibizumab injection and succesfully switched to aflibercept

**DOI:** 10.3205/oc000088

**Published:** 2018-12-13

**Authors:** Ayse Yagmur Kanra, Sevil Ari Yaylali, Ayse Serap Karadag, Aylin Ardagil Akçakaya, Itir Ebru Zemheri

**Affiliations:** 1 Istanbul Sultanbeyli World Eye Hospital, Department of Ophthalmology, Sultanbeyli/Istanbul, Turkey; 2 Istanbul Medeniyet University Göztepe, Training and Research Hospital, Department of Ophthalmology, Istanbul, Turkey; 3 Istanbul Medeniyet University Göztepe, Training and Research Hospital, Department of Dermatology, Istanbul, Turkey; 4 Istanbul Sağlık Bilimleri University Ümraniye Training and Research Hospital, Department of Pathology, Istanbul, Turkey

## Abstract

**Objective:** To report a case of acquired reactive perforating collagenosis (ARPC) triggered by an intravitreal ranibizumab injection that was successfully treated by switching to aflibercept (AFL).

**Methods:** A 73-year-old Caucasian man with an occult choroidal neovascular membrane in the right eye received three-monthly intravitreal ranibizumab injections. Two weeks after the second ranibizumab injection, he complained about a generalized, excessively pruriginous eruption that was further exacerbated by the third injection. On the basis of clinical and histological findings, he was diagnosed with ARPC and treated with narrow band ultraviolet-B (NBUVB) phototherapy.

**Results:** He was subsequently switched to intravitreal AFL injections administered according to a pro re nata regimen. Following NBUVB phototherapy, three additional AFL injections were required. Still, the reactive perforating collagenosis was in remission and the choroidal neovascular membrane was inactive.

**Conclusions:** Our case is the first report of ARPC after ranibizumab injections. Both the skin lesions and the choroidal neovascular membrane were successfully treated after switching to AFL.

## Introduction

Neovascular age-related macular degeneration (n-AMD) is one of the leading causes of blindness in aging individuals throughout the world. Previous studies have shown that the vascular endothelial growth factor (VEGF) is an important contributor to ocular neovascularization and inflammation [1], [2]. There are three available anti-VEGF agents used intravitreally to treat neovascular AMD: bevacizumab (Avastin^®^), ranibizumab (Lucentis^®^) and recently aflibercept (Eylea^®^). Although bevacizumab has been administered off-label since 2005 (because it is only approved for the treatment of advanced solid carcinomas), ranibizumab (RAN) was specifically developed and approved for ocular indications [3]. During pivotal clinical trials, RAN was shown to be an effective and safe treatment for n-AMD owing to its increased potency, enhanced penetration, and decreased likelihood of causing complement-mediated or cell-dependent cytotoxicity [4], [5]. Aflibercept is the most recent US Food and Drug Administration approved treatment of n-AMD (2011). The VIEW 1 and VIEW 2 trials demonstrated similar satisfying efficacy of the two approved drugs in terms of visual and anatomical outcomes. Whereas anti-VEGF agents are generally well tolerated, we present a rare dermatologic side effect after RAN injections that has not previously been described in the literature.

## Case description

**Figure 1:**
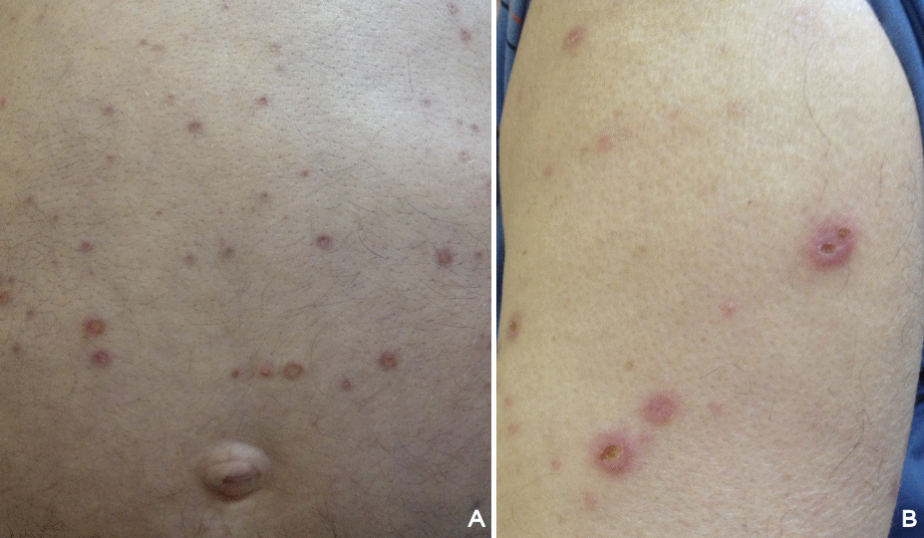
Active and resolving lesions of reactive perforating collagenosis after the third injection of ranibizumab. A) On the trunk, B) on the upper arm. Residual postinflammatory hyperpigmented macules are also seen at the site of prior lesions.

**Figure 2:**
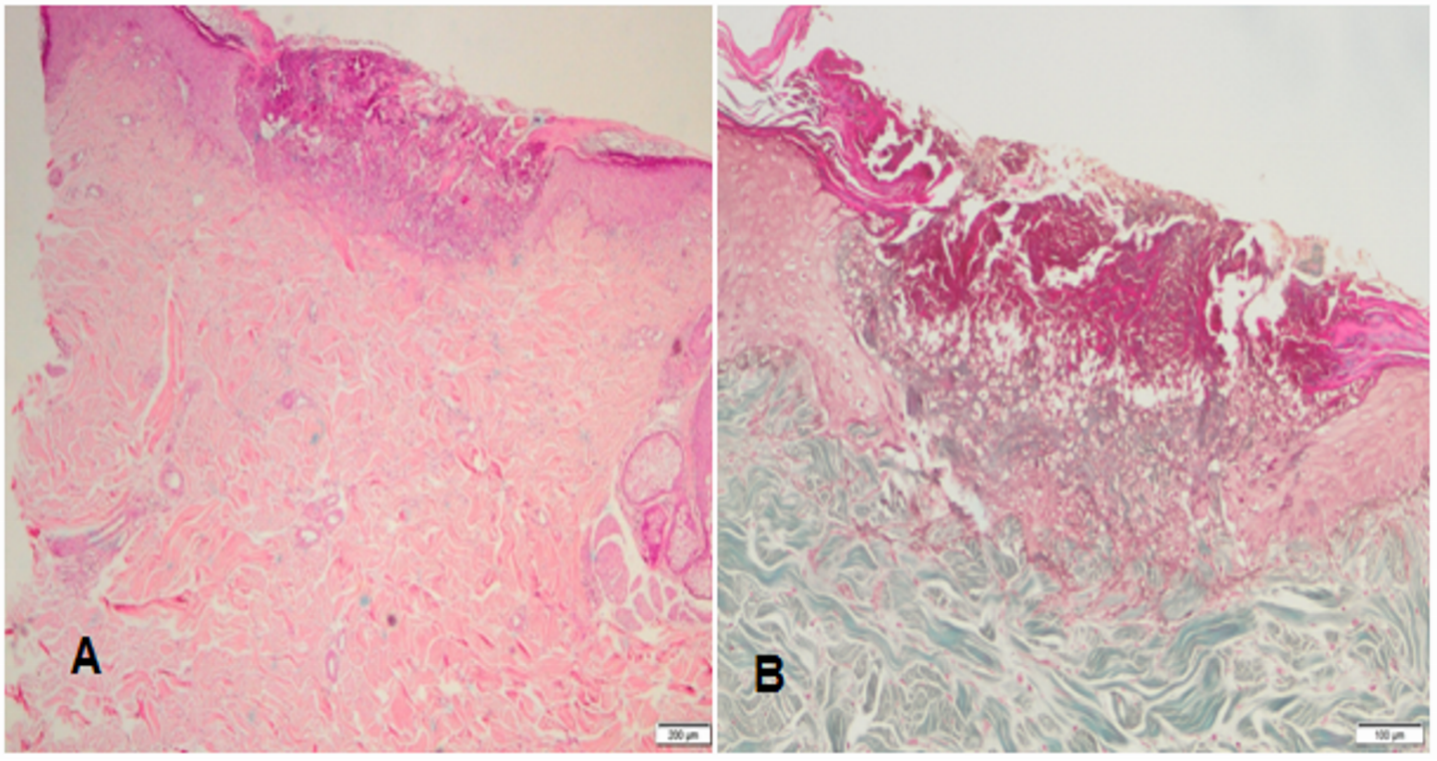
Histopathological features of the reactive perforating collagenosis lesion in our case. A) Cup-shaped depression of the epidermis associated with a keratin plug containing compact ortho- and parakeratosis with granular nuclear debris (H&E). B) Altered collagen fibers in the underlying dermis, with focal extrusion through the epidermis (masson tricrome).

**Figure 3:**
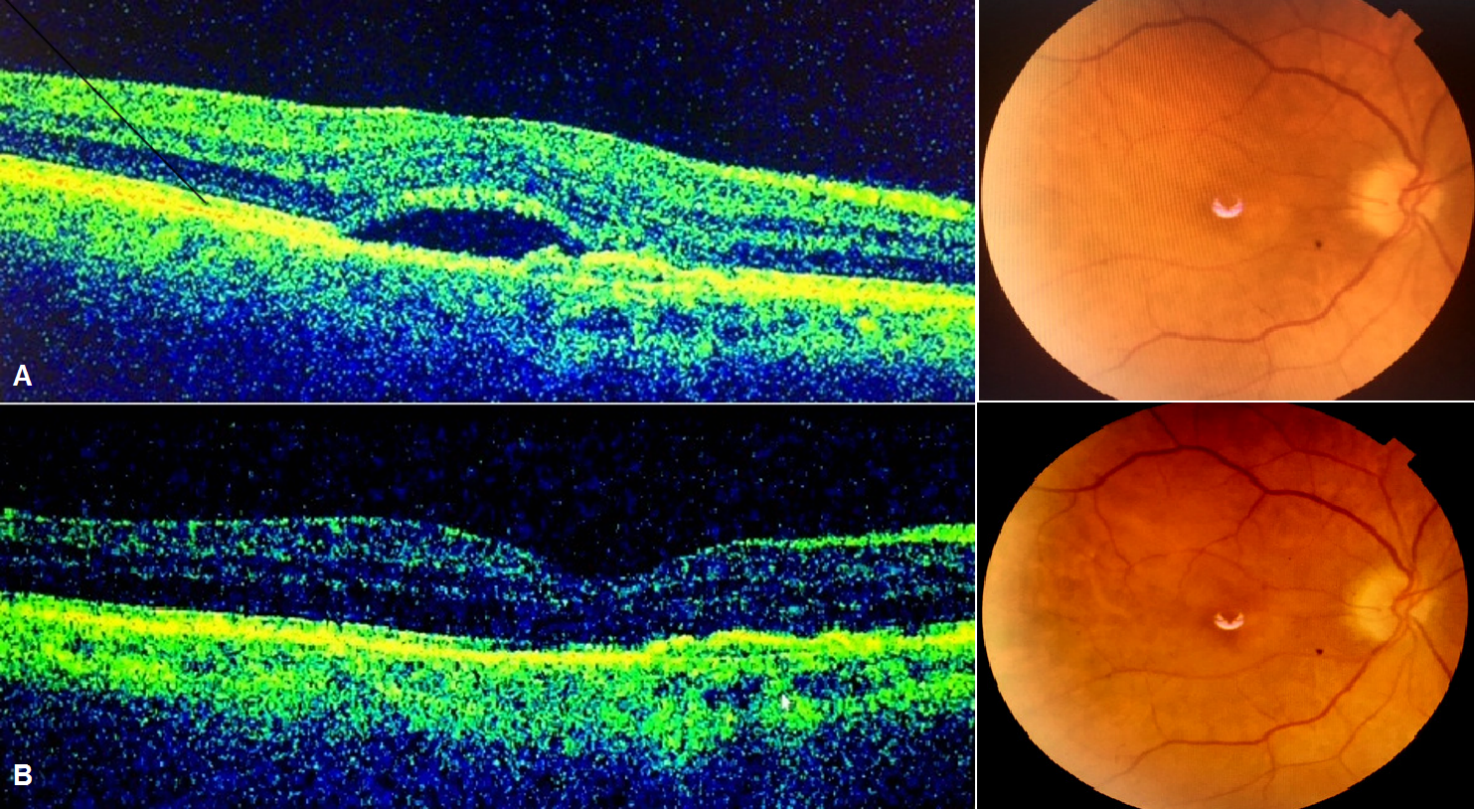
Good anatomical and visual response to AFL injections after switching. A) The subretinal fluid and shallow PED are present at baseline (VA: 20/32). B) No subretinal fluid is seen after the third AFL injection on OCT at the last visit (VA: 20/25). PED: Pigment epithelial detachment. VA: Visual acuity. OCT: Optic coherence tomography.

A 73-year-old Caucasian male with a history of hypertension, diabetes mellitus, and coronary artery disease was referred to our retina department with bilateral vision loss. His medications included klopidogrel, ramipril, amlodipin, atorvastatin, gliclazide, and metformin all of which he had been using for a prolonged period. After fundus fluorescein angiography and optical coherence tomography, he was diagnosed with a ciliary artery occlusion in the left eye with a visual acuity of 20/200. The right eye was found to have an occult choroidal neovascular membrane with subretinal fluid, and so three monthly intravitreal RAN injections were performed. After the third injection, his best-corrected visual acuity (BCVA) improved from 20/32 to 20/25 in association with a decrease in central macular thickness. Two weeks after the second intravitreal RAN injection, he complained about a generalized, excessively pruriginous skin eruption on the trunk, lumbar region, and extensor aspects of the upper and lower extremities. The lesions worsened after the third injection. Laboratory examinations showed mild hyperlipidemia (cholesterol: 241 mg/dl, triglyceride: 256 mg/dl), a blood glucose of 155 mg/dl and HbA1C of 6.6%. In dermatological examination, he had a generalized, erythematous rash with umblicated papules, many of which had ridge-like borders and some contained keratin plugs (Figure 1 (Fig. 1)). A skin biopsy of a nodule demonstrated a cup-shaped depression of the epidermis associated with a keratin plug containing compact ortho- and parakeratosis with granular nuclear debris. Altered collagen fibers were seen in the underlying dermis, with focal extrusion through the epidermis (Figure 2 (Fig. 2)). Based on clinical and histological findings, he was diagnosed with acquired reactive perforating collagenosis (ARPC). A challenge test was considered significant as the rash and pruritus started after the injections. Because of a poor response to topical steroids, 22 sessions of NBUVB therapy were administered to obtain relief from itchy skin. Treatment for CNVM was switched from RAN to aflibercept injection administered according to PRN regimen and 3 doses were required within 13 months. No relapses of the ARPC occurred the subsequent year. Final visual acuity was 20/25 and the central macular thickness was 189 µm (Figure 3 (Fig. 3)).

## Discussion

Reactive perforating collagenosis (RPC) is a rare skin disorder in which degenerated collagen is transepidermally eliminated. The two distinct forms were first reported as a childhood disorder [6] and then as an acquired sporadic form that occurs in association with an underlying systemic disease [7]. The pathogenesis is unclear but it has been speculated that superficial trauma and diabetic microvasculopathy may be responsible for ARPC. In a previous review of nearly 100 patients, 62% were associated with diabetes mellitus (DM) and its complications. In addition, chronic renal failure, malignancies, collagen vascular diseases (lupus, dermatomyositis), viral infections, lung fibrosis, and thyroid dysfunction have been reported regarding to ARPC. Drug-induced ARPC, as occurred in our case, is rarely reported in the literature. Some of these drugs are erlotinib, indinavir, and sirolimus. Treatments for ARPC include topical glucocorticoids, topical and oral retinoids, oral antibiotics, and ultraviolet-B phototherapy although the efficacy is generally variable or unsatisfactory [8]. The lesions are self-healing in many cases but they are intensely itchy and may leave scarring after resolution of the acute phase. Some patients have recurrences, which lead to a chronic course. Emollients and oral antihistamines may relieve itching in the acquired form. Phototherapy is an appropriate treatment for patients with co-existing renal disease and associated pruritus.

In our case, we first excluded the poorly controlled DM with a blood glucose level. Additional testing ruled out renal insufficiency, liver dysfunction, and thyroid disease. Histological analysis was compatible with ARPC and a medication challenge test was considered significant as the rash and pruritus worsened after each RAN injections. 

The incidence of skin rash with intravenous treatment of bevacizumab has been well documented and reported to be as high as 46% and considered to be dose dependent. However, the presence of skin rash as a side effect of intravitreal treatments is a very rare complication. Carneiro et al. claimed that intravitreal bevacizumab significantly reduced VEGF plasma levels until 28 days after the intravitreal injection in patients with neovascular AMD while RAN did not cause a significant plasma VEGF reduction at the same time since RAN is cleared very quickly from the systemic circulation [9]. It is speculated that patients with lower systemic levels of VEGF-A are at higher risk for systemic complications because VEGF is important to maintain adequate perfusion throughout the body [10]. Aflibercept is a decoy receptor binding to VEGF-B and the placental growth factor, in contrast to ranibizumab and bevacizumab, which only bind to VEGF-A. Aflibercept has not been a well documented drug for lowering plasma VEGF levels. Furthermore, measurement of real plasma free VEGF can be a challenge, as platelet rupture and/or activation can provide the release of intracellular VEGF and result in rising VEGF levels. Comprehensive review of 10 phase II and III trials of intravitreal aflibercept in retinal disease showed that rates of selected ocular and systemic adverse events were similar to those of controls.

## Conclusion

ARPC is most commonly seen in patients with uncontrolled DM but our patient had regulated diabetes and his problems occurred after RAN injections. To the best of our knowledge, this is the first report of ARPC after RAN injections that was successfully treated with NBUVB therapy and effective switching to aflibercept.

## Notes

### Competing interests

The authors declare that they have no competing interests.
